# Therapeutic Potential of Tolerogenic Dendritic Cells in IBD: From Animal Models to Clinical Application

**DOI:** 10.1155/2013/789814

**Published:** 2013-11-12

**Authors:** Raquel Cabezón, Daniel Benítez-Ribas

**Affiliations:** ^1^Fundació Clinic, Hospital Clínic i Provincial and Centre Esther Koplowitz, 08036 Barcelona, Spain; ^2^Department of Experimental Gastroenterology, Centro de Investigación Biomédica en Red de Enfermedades Hepáticas y Digestivas (CIBERehd), Hospital Clínic i Provincial and Centre Esther Koplowitz, Carrer Roselló 149-153, 08036 Barcelona, Spain

## Abstract

The gut mucosa undergoes continuous antigenic exposure from food antigens, commensal flora derived ligands, and pathogens. This constant stimulation results in controlled inflammatory responses that are effectively suppressed by multiple factors. This tight regulation, necessary to maintain intestinal homeostasis, is affected during inflammatory bowel diseases (IBD) resulting in altered immune responses to harmless microorganisms. Dendritic cells (DCs) are sentinels of immunity, located in peripheral and lymphoid tissues, which are essential for homeostasis of T cell-dependent immune responses. The expression of a particular set of pathogen recognition receptors allows DCs to initiate immune responses. However, in the absence of danger signals, different DC subsets can induce active tolerance by inducing regulatory T cells (Treg), inhibiting inflammatory T helper cell responses, or both. Interestingly, several protocols to generate clinical grade tolerogenic DC (tol-DCs) *in vitro* have been described, opening the possibility to restore the intestinal homeostasis to bacterial flora by cellular therapy. In this review, we discuss different DC subsets and their role in IBD. Additionally, we will review preclinical studies performed in animal models while describing recent characterization of tol-DCs from Crohn's disease patients for clinical application.

## 1. Introduction

The gut mucosa is continuously exposed to external food antigens and pathogens and to commensal flora microorganisms, mostly bacteria and fungi. This constant antigenic stimulation results in controlled inflammatory responses that are effectively suppressed by multiple nonimmune and immune factors. The intestinal immune system is capable of distinguishing between invasive organisms and harmless antigens. The host response to the intestinal microbiota can be categorized into three important categories: (1) the intestinal epithelium, which can efficiently modulate immune response by secreting inflammatory mediators, recruiting DCs and presenting antigens to T lymphocytes, (2) the innate immunity, including anatomical barriers, secretory molecules, and cellular components, that initiate the nonspecific immune response, and (3) the adaptive immunity, which is driven by B and T lymphocytes, responsible for antigen specific immune responses. This tight regulation, necessary to maintain intestinal homeostasis, is altered in IBD, resulting in uncontrolled immune responses to harmless microorganisms. Adaptive immunity is the most putative driver of tissue damage seen in IBD patients, although innate immune responses are definitively a prerequisite for the excessive activation of adaptive immunity [1]. Several studies have proposed that the inappropriate activation of DCs may contribute to the pathogenesis of IBD [2].

DCs are the most potent antigen-presenting cells linking innate and adaptive immune responses. Located in peripheral and lymphoid tissues, DCs are sentinels of the immune system recognizing and translating pathogenic or harmless signals into immunogenic or tolerogenic responses, respectively. DCs are especially well equipped to continuously sample these tissues for the presence of pathogenic microorganisms, and their detection relies on the recognition of conserved molecular structures, known as pathogen-associated-molecular-patterns (PAMPs) via pattern recognition receptors (PRRs). DCs orchestrate adaptive immune responses linking innate recognition of pathogens and driving and polarizing naïve T cells activation. Due to their physiological properties and the availability of clinical grade reagents, DCs have been safely and successfully used in clinical trials aimed at stimulating an efficient immune response against tumors or infectious diseases [3, 4]. However, only a few recent studies have taken advantage of their specific tolerogenic properties to treat Type 1 diabetes [5] and rheumatoid arthritis patients [6]. Both studies have taken place in the last two years; thus, it is still too early to draw any conclusion in relation to their clinical efficacy. The majority of clinical studies to date have been carried out with *ex vivo* generated monocyte-derived DCs taking advantage of their plasticity. Several protocols to generate tol-DC have been described using different agents, including glucocorticoids such as dexamethasone [7, 8], mycophenolic acid [9], vitamin D3 (1*α*,25-dyhydroxyvitamin D3) [10], retinoic acid (RA), the combination of dexamethasone and vitamin D3 [11, 12], or rhIL-10 [13], which have been used to render DCs resistant to maturation. Therefore, *ex vivo* generated tol-DCs are considered as therapeutic vaccines to reestablish antigen-specific tolerance in autoimmune disorders. The aim of this review is to discuss DC subsets and their role in tolerance induction, preclinical studies in animal models of colitis, and our recent findings on tol-DC generation and characterization in humans for clinical applications in Crohn's disease patients.

## 2. Human DC Subsets

Several subsets of circulating DCs have been defined in humans based on the lack of expression of typical lineage (CD3, CD19/CD20, CD14, and CD56) negative markers (lin^−^) and high levels of MHC class II (HLA-DR) positive cells [14]. Furthermore, a number of positive DC markers have been used to identify different DC subsets. Plasmacytoid DCs (CD11c^−^) are identified by the expression of BDCA-2 and BDCA-4 plus CD123, whereas myeloid DCs (CD11c^+^) can be further subdivided in BDCA-1 (CD1c^+^) and BDCA-3 (CD141^+^) positive cells. Although evidence suggests the involvement of a particular DC subset in tolerance induction, it is now accepted that different DC subsets participate in immunity or tolerance showing functional maturation and plasticity depending on environmental signals received. However, recent advances have helped to associate the DC subsets to functional specialization. This functional specialization is linked to the different expression between DCs subsets of PRRs like toll-like receptors (TLR), C-type lectins, and the cytoplasmic NOD family proteins, as well as RIG-I and MDA-5 molecules. Whether this functional specialization is linked to different aspects of tolerance induction is an issue to be formally established. 

The gastrointestinal immune system is continuously exposed to potent stimuli from commensal bacteria and food. A specialized network of immune cells is organized in the mucosa in order to maintain immunologic tolerance. How DCs regulate immune response in the gut has been deeply investigated, and several DCs subsets and their function have been identified in mice. But whether these subsets are equivalent in humans needs to be further studied (reviewed by Mann et al. [15]). It is well known that DCs in the gut are generally hyporesponsive [16] and have the ability to imprint homing properties on T cells. Although defining cell markers to identify intestinal DCs is controversial, there are different strategies to identify human DCs in the mucosa. The most common one is by negative lineage of CD3, CD14, CD16, CD34, and expression of HLA-DR^+^. These cells are often mistaken as macrophages, and some authors prefer to differentiate both subsets by function and not by cell markers. Interestingly, CD103^+^ DCs have tolerogenic properties and share some functional aspects with murine CD103^+^ DCs. These “tolerogenic” DCs promote Treg differentiation [16] and produce RA [17] and indoleamine 2,3-dioxygenase (IDO) [18], molecules that can drive Tregs and are known to be involved in the induction of tolerance in the gut. The ability of CD103^+^ DCs to produce IL-10 and the lack of IL-12, together with the low expression of CD40, TLR2, and TLR4, make these cells suitable to be defined as the main regulators of immune tolerance in the intestinal tract. However, research in this area is very difficult and a lot more needs to be elucidated regarding human DC subsets and tolerance in the gut.

## 3. Mechanisms to Induce Tolerance

**Figure 1:**
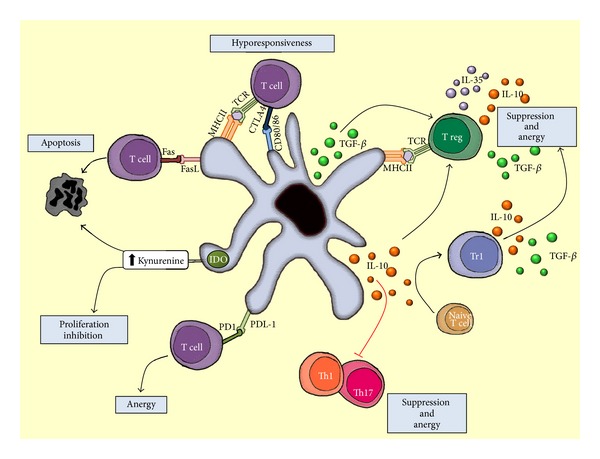
Summary of different mechanisms to induce tolerance by DCs; Treg and Tr1 cells generation, suppression of T effector cells, inhibition of proliferation, apoptosis induction, T cell anergy, and hyporesponsiveness.

The functional properties of DCs are dependent on their maturation status. Due to the lack of expression of costimulatory molecules and MHCII, tol-DCs are able to induce T cell anergy, preventing T cell activation. It has been described that DCs suboptimal antigen presentation, combined with the expression of IDO or FasL (CD95L) leads to inhibition of T cell proliferation and T cell deletion [19] (Figure 1). The induction of Treg and Type 1 regulatory T cells (Tr1) by DCs is another mechanism to induce peripheral tolerance [20, 21]. The mechanisms by which DCs regulate immune responses, tolerance, and lamina propria homeostasis against commensal flora have not been fully elucidated. The immunosuppressive cytokine IL-10 is a crucial mediator of tolerance in the gut and it has a nonredundant role in limiting inflammatory responses in the intestine. IL-10 can act on a variety of immune cells and its secretion is certainly involved in Tregs and Tr1 induction as well as regulating the local inflammatory immune response via antigen-presenting cells [22–24]. Indeed, IL-10 is very important in maintaining intestinal homeostasis as revealed by the spontaneous chronic inflammatory disease (similar to Crohn's disease) that IL-10 knockout mice develop. In addition, a severe form of Crohn's disease (with early-onsets of enterocolitis, involving hyperinflammatory immune responses in the intestine) in infants was associated with IL-10 receptor mutations in two unrelated consanguineous families [25]. IL-10 signaling directly suppresses Th17 and Th17^+^Th1^+^ cells in mice with established colitis [26]. All these features together make tol-DCs suitable to create an immunosuppressive environment that can potentially induce tolerance in the neighboring tissue. 

## 4. Plasmacytoid Dendritic Cells and Tolerance

Plasmacytoid DCs (pDC) appear to play an important role in the regulation of tolerance induction [27], transporting self-antigens from peripheral tissues in the thymus contributing to the inactivation of autoreactive T cells and induction of Tregs [28, 29]. While present in tissues at very low numbers in the healthy steady-state, pDCs accumulate in lymphoid and nonlymphoid tissues under pathological or inflammatory conditions. In addition, the role of pDCs in controlling the intestinal homeostasis is largely unknown. Interestingly, liver and spleen pDCs express higher levels of NOD2 than conventional myeloid DCs (mDCs) and pDC are able to detect and respond to muramyl dipeptide (MDP). NOD2 ligation reduces IL-12, IL-6, and TNF-a production by pDC, in the presence or absence of either LPS or CpG stimulation [30]. Aberrant accumulation of pDCs in MLN and inflamed mucosa of IBD patients compared to controls has been shown [31]. Furthermore, highly purified pDCs from patients produced high levels of proinflammatory cytokines and showed an activated phenotype. However, IFN-*α* secretion induced by CpG-A was impaired in pDCs from IBD patients [31]. Another report showed that IBD patients lack circulating immature blood DCs (both DC subsets, myeloid, and plasmacytoid) during flares, which possibly migrate to the gut. An aberrant response to microbial surrogate stimuli suggests a disturbed interaction with commensals [32]. It has been recently shown that CCR9 expression in pDCs can home to the gut [33] and induce potent Treg responses that have a significant therapeutic effect in a model of intestinal Graft Versus Host Disease [34]. It is important to highlight that the clinical benefit of G-CSF therapy in Crohn's disease patients is thought to be related to its ability to induce IL-10-mediated regulatory functions, associated possibly with increased pDCs numbers in the inflamed gut [35]. However, a direct correlation between pDCs and the clinical benefit has not been established yet. Despite their reported role in the pathogenesis of certain autoimmune diseases, such as SLE or psoriasis, understanding pDC function in the pathogenesis of human diseases has just begun.

## 5. Role of Tol-DCs in IBD Animal Models

Experimental data generated in murine models of colitis are highly promising especially relating to the ability of tol-DCs to prevent, reverse, or ameliorate established colitis [12, 36, 37]. In a model of TNBS-induced colitis, which closely parallels the immune activation in Crohn's disease, injection of tol-DCs treated with Vasoactive Intestinal Peptide (VIP) [38] significantly ameliorated the clinical and histopathology severity of colitis in mice. An important aspect of this study was the route of administration of the DCs; the authors clearly show that by intraperitoneal administration DCs gain access to mesenteric lymph nodes, where the most important antigen presentation and activation of Th1/Th17 cells takes place [39]. In addition, different types of tol-DCs generated with a combination of dexamethasone plus vitamin D3 [12] or loaded with enterobacterial extract [36] were able to prevent the colitis induction. Several other animal models have revealed the therapeutic role of tol-DCs in preventing and ameliorating IBD in an antigen-specific way [37]. However, the current challenge is to bring this tol-DC therapy to the clinic for human patients. Several issues must be overcome such as the difference between IBD-induced animal models (reviewed by Neurath [40]) and the human disease, or the functional differences between mouse and human DCs. In summary, those promising results in rodents await to be translated into the human application.

## 6. Therapeutic Application of Tol-DCs in Crohn's Disease Patients

**Figure 2:**
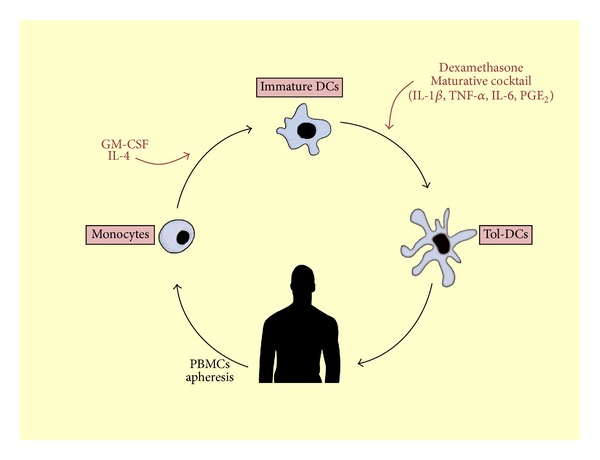
General scheme: dendritic cell therapy for Crohn's disease patients. Isolated monocytes are cultured and differentiated into DCs by adding IL-4 and GM-CSF to the media. At day 3, addition of dexamethasone induces the tolerogenic profile, and at day 6, addition of the maturation cytokine cocktail potentiates the tolerogenic properties.

We have developed a protocol to produce tol-DCs under clinical grade conditions for the treatment of Crohn's disease patients (Figure 2) by conditioning monocyte-derived DC with dexamethasone at day 3, together with 24 hours maturation with a cytokine cocktail (IL-1*β*, IL-6, and TNF-*α*) plus PGE_2_ [41]. Compared to mature and immature DCs, our tol-DCs produced higher levels of IL-10, even in response to gram-negative bacteria or synthetic LPS, with low or undetectable levels of IL-12p70, IL-23, or TNF-*α*. In addition, tol-DCs phenotype was consistently semimature with intermediate expression of costimulatory receptors (CD80 and CD86), low levels of CD83 and MHC class II, and the ability to inhibit T cell responses. It has been shown that DC activation with LPS or the clinical grade TLR4 ligand MPLA enhances tol-DCs migratory properties and antigen presentation capabilities [42]. Interestingly, even though the fact that isolated monocytes from Crohn's disease patients are in an enhanced proinflammatory environment [32], we showed that these cells from Crohn's disease patients can be educated towards tolerogenic phenotype. These results are in line with studies in other immune-based diseases like rheumatoid arthritis or multiple sclerosis [43, 44]. This is a key aspect for considering this DC-based treatment as a therapeutic option in IBD, because it might have occurred that genetic variants conferring susceptibility for Crohn's disease or the proinflammatory environment might alter the biology of DCs.

## 7. Lack of Crohn's Disease Associated Antigen

DC-based therapies are envisaged to inhibit antigen-specific T cell responses, and the appropriated antigen selection to load DCs is under intensive research. Although humoral response against antigens derived from microbiota has been described in Crohn's disease patients, for example, elevations in anti-Saccharomyces cerevisiae antibodies (ASCA) in 49–60% of cases [45], no T lymphocyte Crohn's disease-specific antigen has been properly identified. Although the disease is associated with a high inflammatory component, mainly corresponding to Th1 and Th17 T cells [38, 39], the antigenic specificity of these cells remains to be investigated. Interestingly, commensal-specific T cell responses are detected during mouse model of intestinal inflammation with *Toxoplasma gondii* infection [46, 47]. It is tempting to speculate that commensal specific T cells may represent an important component of the IBD, although much remains to be understood about this issue. In animal models, Yamanishi et al. [37] identified a specific protein, carbonic anhydrase I (CA I), specifically involved in the IBD pathogenesis. Interestingly, the authors demonstrate the role of CA I loaded tol-DCs in preventing the induction of colitis via Tregs. Pedersen et al. administered DCs pulsed with enterobacterial extract to suppress development of colitis [36]. However, other authors have demonstrated tolerance induction in colitis model without antigens [12, 38]. This mechanism would involve the generation of DCs secreting regulatory cytokines (TGF-*β* and IL-10) and expressing inhibitory receptors that might overcome the necessity of a known antigen. This “transtolerance” may result in the generation of a specific regulatory response helping to restore the mucosal homeostasis. 

## 8. Summary

DCs are powerful therapeutic tools to modify the immune response and restore the immune tolerance in Crohn's disease patients and other autoimmune diseases. An alternative to manipulate the different subsets of intestinal DC function is the *in vitro* generation of tol-DCs. Methods to obtain these cells in sufficient amounts have been developed. Tol-DCs may represent a new therapeutic strategy for Crohn's disease, where the alterations of the finely tuned balance between the immune system and the microflora result in disease. Several reports have indicated the therapeutic effect of tol-DCs in inhibiting IBD induction in animal models. These results highlight the importance of DCs in the intestinal homeostasis control and open new avenues for an innovative therapeutic indication for human patients.
